# Supplementary material for: Identification and functional analysis of non-coding regulatory small RNA FenSr3 in Bacillus amyloliquefaciens LPB-18
Source: PeerJ. 2023 May 15;11:e15236. doi: 10.7717/peerj.15236 (PMC10194069; doi:10.7717/peerj.15236)
Supplement: Supplemental Information 4 [file peerj-11-15236-s004.zip › KO/CK-vs-T1_map/map05219.html]

KEGG PATHWAY: Bladder cancer - Reference pathway


|  |  |
| --- | --- |
| **Bladder cancer - Reference pathway** |  |

[
Pathway menu
| Organism menu
| Pathway entry
| Show description
| User data mapping
]

The urothelium covers the luminal surface of almost the entire urinary tract, extending from the renal pelvis, through the ureter and bladder, to the proximal urethra. The majority of urothelial carcinoma are bladder carcinomas, and urothelial carcinomas of the renal pelvis and ureter account for only approximately 7% of the total. Urothelial tumours arise and evolve through divergent phenotypic pathways. Some tumours progress from urothelial hyperplasia to low-grade non-invasive superficial papillary tumours. More aggressive variants arise either from flat, high-grade carcinoma in situ (CIS) and progress to invasive tumours, or they arise de novo as invasive tumours. Low-grade papillary tumors frequently show a constitutive activation of the receptor tyrosine kinase-Ras pathway, exhibiting activating mutations in the HRAS and fibroblast growth factor receptor 3 (FGFR3) genes. In contrast, CIS and invasive tumors frequently show alterations in the TP53 and RB genes and pathways. Invasion and metastases are promoted by several factors that alter the tumour microenvironment, including the aberrant expression of E-cadherins (E-cad), matrix metalloproteinases (MMPs), angiogenic factors such as vascular endothelial growth factor (VEGF).

|  |  |  |
| --- | --- | --- |
| Reference pathway | 184% 150% 122% 100% 82% 67% 55% | 图片下载 |
